# Rolling bearing fault diagnosis method based on dual attention multi-scale convolutional network

**DOI:** 10.1371/journal.pone.0358253

**Published:** 2026-09-21

**Authors:** Yamei Zhang, Xiaohu Liu

**Affiliations:** 1 School of Intelligent Science and Engineering, Xi’an Peihua University, Xi’an, China; 2 Trine Engineering Institute, Shaanxi University of Technology, Hanzhong, China; Beijing Institute of Technology, CHINA

## Abstract

To address the significant decline in the accuracy of rolling bearing fault diagnosis under variable working conditions and strong noise, which is caused by feature distribution shift and fault feature submersion, this paper proposes a bearing fault diagnosis method based on the Dual Attention Multi-Scale Convolutional Neural Network (DAMSCNN). First, a parallel multi-scale convolution module with multiple kernel sizes extracts fault features under different receptive fields, overcoming the limitation of fixed receptive fields in single-scale convolution. Second, a cascaded channel-spatial dual attention mechanism is designed following a progressive weighting strategy. A residual connection is embedded in the channel attention branch to highlight fault-sensitive channels and suppress redundant ones. The spatial attention branch fuses global average pooling and max pooling to strengthen fault impulse regions and suppress interference from working condition fluctuations and background noise. Finally, the weighted features are classified by a Softmax classifier. Experiments on the CWRU bearing dataset demonstrate that DAMSCNN achieves a peak diagnostic accuracy of 99.69% in cross-condition tasks and maintains accuracy above 92% under −5 dB SNR, outperforming BiTCN-Transformer, MTFC-T, and CTCA in accuracy, recall, and F1-score, while also offering lower computational cost and faster inference speed.

## 1. Introduction

With the continuous advancement of science and technology, modern large-scale rotating machinery has been evolving toward intelligence, high precision, and high integration. As critical supporting components of rotating equipment, rolling bearings directly determine the operational stability and overall service performance of mechanical systems. Accordingly, bearing fault diagnosis has become a core research topic in the field of mechanical condition monitoring and fault diagnosis [1]. Unexpected bearing faults easily lead to equipment shutdown and production suspension, resulting in considerable economic losses and even serious personal safety accidents. Therefore, accurate and reliable bearing fault diagnosis carries important theoretical significance and engineering practical value for ensuring the safe and stable operation of industrial equipment and preventing potential safety hazards [2,3]. Driven by the rapid iteration of computer technologies and artificial intelligence algorithms, bearing fault diagnosis has evolved from traditional empirical judgment to intelligent data-driven diagnosis. Early diagnostic methods primarily relied on manual experience and expert knowledge, which suffered from low efficiency and limited generalization capability. With the advancement of data-driven theories, mechanism modeling and shallow machine learning approaches have been extensively adopted for bearing fault diagnosis. Classical machine learning algorithms, such as K-Nearest Neighbors (KNN), Support Vector Machines (SVM), and Random Forest, can achieve satisfactory diagnostic accuracy under simple and stable operating conditions [4]. Nevertheless, these methods heavily rely on manual feature engineering and expert experience, and their diagnostic performance degrades significantly under complex and variable working conditions, leading to poor generalization and robustness in practical industrial applications [5]. In contrast, deep learning-based fault diagnosis methods implement end-to-end automatic feature extraction and intelligent classification. These methods effectively overcome the limitations of manual feature selection and deliver superior diagnostic performance under steady operating conditions. However, actual industrial bearing operation scenarios commonly involve variable speeds, fluctuating loads, and strong background noise, which generate complex, coupled, and non-stationary vibration signal components [6,7]. Specifically, variable-speed conditions induce significant temporal and spectral variations in fault impulse characteristics, presenting dual-domain distribution features [8]. Variable-load conditions lead to dynamic fluctuations in the spectral amplitude of vibration signals, while abrupt load impacts introduce extra impulse components. These components alias and couple with inherent bearing fault characteristics, further increasing the difficulty of effective fault feature extraction [9]. Such complex operating condition fluctuations cause distribution discrepancies between training and test datasets, violating the independent and identically distributed assumption of conventional deep learning models and resulting in severe domain shift. Accordingly, traditional deep learning methods exhibit degraded diagnostic accuracy and poor generalization robustness under variable industrial environments, which greatly restricts their large-scale industrial deployment [10]. Notably, beyond the field of rolling bearing fault diagnosis, convolutional neural networks have exhibited universal effectiveness in complex feature extraction tasks across diverse engineering domains. For instance, Li et al. proposed weight clustered-convolutional neural network-long short-term memory (WC-CNN-LSTM), the method can achieve high accuracy SOT and SOC estimation with small model sizes [11]. Xu et al. proposed a dynamic range compression dual-domain attention network (DRC-DFANet) for real-time and high-precision enhancement of tunnel images, this method can improve the image acquisition capabilities of transportation visual systems [12]. These cross-domain applications further substantiate the rationality of convolutional networks for processing complex, non-stationary signals.

To mitigate the performance degradation induced by complex and variable operating conditions, numerous studies have integrated traditional signal processing algorithms with deep learning techniques to achieve adaptive bearing fault diagnosis. Typical signal processing methods, including order tracking and time-frequency analysis, have been widely combined with deep learning frameworks to strengthen feature extraction ability under varying working conditions [12–14]. Representative achievements include the integration of convolutional neural networks with order tracking for bearing fault identification under variable speeds [15], the combination of deep residual networks with the Short-Time Fourier Transform (STFT) for variable-condition fault diagnosis [16], and the fusion of Continuous Wavelet Transform (CWT), residual expansion networks, and denoising autoencoders to adapt to complex operating scenarios [17]. Although such hybrid methods can improve diagnostic performance to a certain degree, they are generally limited by low computational efficiency and insufficient adaptability to varying working conditions. In recent years, transfer learning and multi-scale intelligent diagnosis theories have emerged as research hotspots for bearing fault diagnosis under complex working conditions, providing effective solutions for domain adaptation and cross-condition diagnostic tasks [18]. Yin et al. proposed a multi-scale transfer learning framework to enhance the cross-domain adaptive feature learning capability of diagnosis models [19]. Zheng et al. combined the improved CEEMDAN algorithm with a CNN-BiGRU hybrid model to improve the fault diagnosis accuracy of industrial bearings under complex conditions [20]. Wang et al. established a multi-scale convolutional attention network based on multi-modal time-series transformation to extract multi-dimensional fault features [21]. Yu et al. developed a dedicated diagnosis method for wind turbine bearings to achieve reliable fault feature extraction under strong noise interference [22]. Luan et al. integrated wavelet packet transform and the CEEMDAN algorithm to suppress noise and extract weak fault characteristic frequencies [23]. Despite these promising advances, several challenges remain to be addressed, such as insufficient capability in extracting domain-invariant features across different working conditions and unsatisfactory model interpretability, which necessitates further in-depth research.

To address the above challenges, this paper proposes a novel multi-scale convolutional network integrated with a dual attention mechanism for generalized cross-domain bearing fault diagnosis. The main research contributions of this work are summarized as follows:

(1) To address the limitation that traditional single-scale convolutional networks suffer from fixed receptive fields and fail to fully characterize the multi-scale fault information of bearing vibration signals under variable working conditions, a parallel multi-scale convolutional feature extraction module is constructed. Multi-size convolution kernels are adopted to synchronously extract fault features under different receptive fields, so as to achieve comprehensive capture of fault information and enhance the model’s capability of characterizing complex fault features.
(2) Adopting a progressive weighting strategy of “channel screening first, spatial enhancement second”, a two-stage cascaded channel-spatial dual attention mechanism is designed. In this mechanism, a residual connection structure is embedded in the channel attention branch to adaptively assign channel weights, selecting feature channels strongly correlated with bearing faults and suppressing redundant channels. The spatial attention branch generates a spatial weight map by fusing global average pooling and max pooling operations, to highlight fault impulse regions and suppress noise and working condition-coupled interference components. Through the cascaded synergy of the two-stage attention, adaptive enhancement of domain-invariant fault features is realized, which improves the generalization robustness of the model under variable working conditions and strong noise environments.

### 2. Fault diagnosis method based on DAMSCNN

In complex and dynamic practical working environments, deep learning-based bearing fault diagnosis models often face significant challenges in efficiently extracting fault features that can both accurately identify bearing health status and remain robust to working condition fluctuations. Since bearings mostly operate under variable load and variable speed conditions, the collected vibration signals exhibit multi-scale distribution, noise coupling, and inconsistent data distribution. These characteristics lead to domain shift, degraded generalization performance, and reduced diagnostic accuracy in traditional models. To address these issues, this paper proposes a rolling bearing fault diagnosis method based on the Dual Attention Multi-Scale Convolutional Neural Network (DAMSCNN). Taking raw vibration signals as input, the method follows an end-to-end pipeline consisting of multi-scale feature mining, dual-dimensional attention weighting, and feature fusion classification, enabling high-accuracy and stable fault diagnosis under variable working conditions and strong noise environments.

To extract multi-scale fault features, suppress sensitivity interference caused by working condition fluctuations, and produce stable diagnostic results in bearing fault diagnosis, this paper constructs a deep learning model that integrates multi-scale convolution with a dual attention mechanism. The multi-scale convolution structure is adopted to match the multi-scale distribution characteristics of bearing fault signals, alleviating the limitation of incomplete fault feature extraction caused by the single receptive field of traditional convolution. A two-stage attention mechanism covering channel and spatial dimensions is introduced to apply adaptive weighting to features, highlighting fault information while suppressing interference induced by working condition variations and noise. Through deep fusion of multi-scale features, accurate bearing fault diagnosis is achieved under complex environments with variable working conditions and strong noise. The specific implementation steps of the proposed method are as follows:

**Step 1:** Perform sample interception and normalization on the vibration signals from the CWRU bearing dataset to unify the input format of the model.

**Step 2:** Construct a multi-scale convolution module with parallel multi-size convolution kernels to perform convolution operations on the preprocessed signals. This module simultaneously extracts local fine-grained and global coarse-grained fault features, covering fault information across different receptive fields, and addressing the challenge of extracting fault features with varying scales under variable working conditions.

**Step 3:** Extract multi-scale features of bearing vibration signals through convolutional layers, then implement global compression and channel weight learning via the channel attention mechanism. The features output by the channel attention module are subsequently fed into the spatial attention module, which dynamically assigns adaptive weights in the spatial dimension of feature maps, thereby realizing feature optimization and adjustment in both the channel and spatial dimensions.

**Step 4:** Process the dual attention-weighted multi-scale features through two-dimensional convolution for deep feature mining, followed by batch normalization, max pooling, and Dropout for overfitting prevention. The processed features are then mapped into one-dimensional feature vectors via the Flatten layer, generating discriminative features with certain robustness against working condition fluctuations.

**Step 5:** Feed the deeply fused features into the fully connected layer for nonlinear mapping and feature transformation. Finally, output the probability values corresponding to each bearing fault type through the Softmax classifier, and select the fault category with the maximum probability as the final diagnostic result.

## 3. Dual attention multi-scale convolutional neural network

### 3.1. Convolutional neural network

Convolutional Neural Network (CNN) is a class of feedforward neural networks with convolution operations as its core structure. Its key functional and structural characteristics are outlined as follows:

Local connectivity: To support local feature learning, only local connections exist between neurons in adjacent layers during forward propagation.

Weight sharing: Each convolution kernel in the network operates repeatedly across the entire receptive field with identical shared parameters.

Pooling operations and multi-layer architecture: Pooling is essentially a form of downsampling operation. It can significantly reduce the computational cost of data processing while effectively preserving the core useful information in the signal. By employing multiple convolution kernels, the network generate diverse features and thus obtain richer feature representations [24,25].

**Fig 1:**
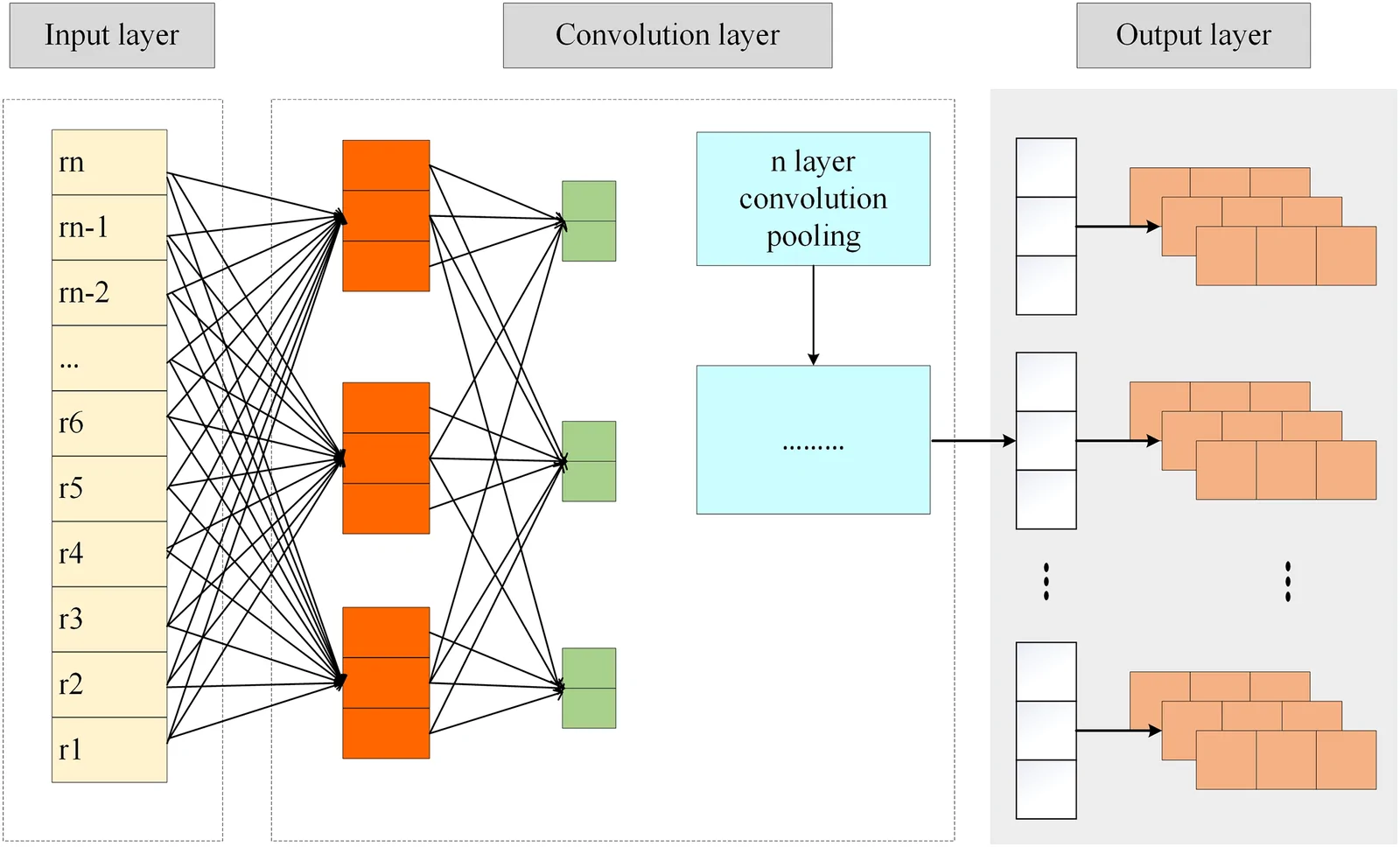
The structure of CNN.

The architecture of the CNN is illustrated in Fig 1. Convolution and pooling operations constitute the main components of the network, which are primarily responsible for feature extraction and parameter compression. The mathematical expression of the convolution operation is given as follows:


$$\begin{array}{c} y_{j}=f\left[\sum_{i=1}^{n}(x*w_{i})+b_{i}\right] \end{array}$$ (1)

where x is the input; $y_{j}$ is the output of the *j*-th; n is the number of convolution kernels; * is a convolution operator; $w_{i}$ is the *i*-th convolution kernel; $b_{i}$ is the bias term; f() is the ReLU activation function.

Vibration signals of rolling bearings collected in practice are inevitably contaminated with fault-irrelevant interference, such as environmental noise and rotational speed fluctuations. Such interference undermines the network model’s ability to learn fault features, leading to a decline in diagnostic accuracy. Therefore, it is necessary to integrate an attention mechanism into the rolling bearing fault diagnosis model to effectively enhance its focus on fault features and improve diagnostic accuracy.

### 3.2. Multi-scale feature extraction module

**Fig 2:**
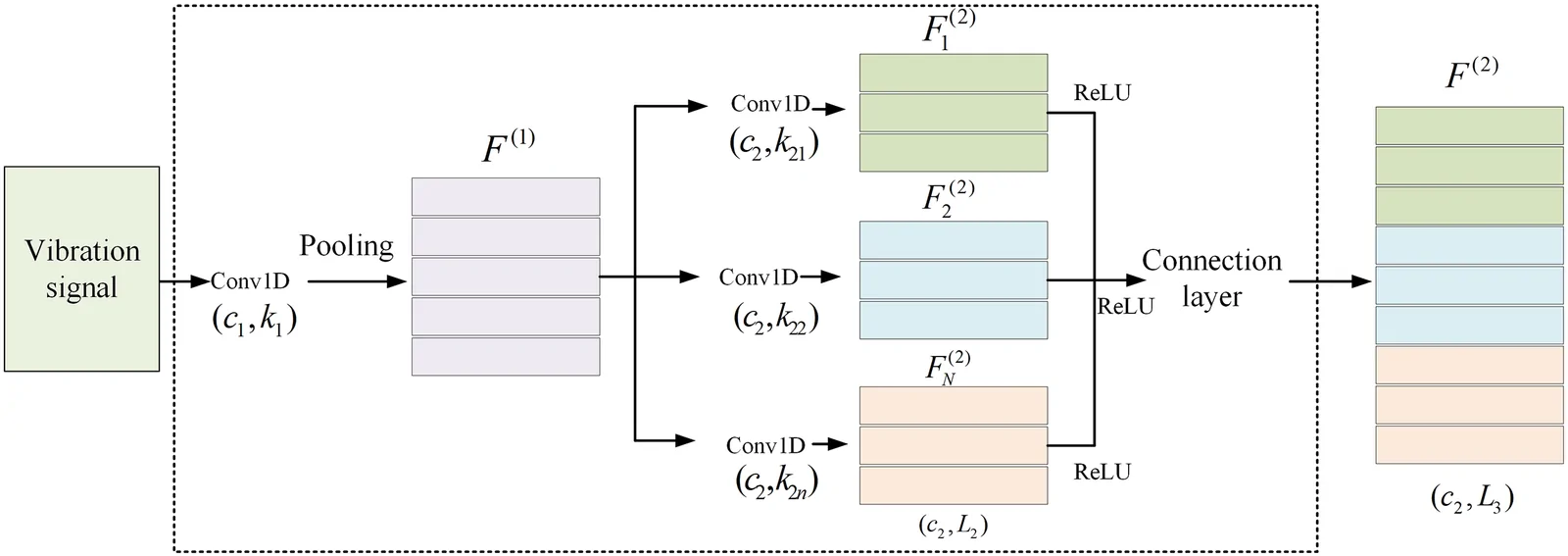
The structure of multi-scale feature extraction module.

The structure of multi-scale feature extraction module is illustrated in Fig 2.

Wide convolution kernels are adopted to extract features from raw vibration signals. Acting as low-pass filters, such kernels can effectively suppress high-frequency noise and mitigate data overfitting in the convolved features. The mathematical formulation of the convolution operation is expressed as follows:


$$\begin{array}{c} F^{\left(1\right)}=\left[f_{\text{1}}\left(k_{\text{1}}*x+b);\cdots ;f_{c_{\text{1}}}(k_{\text{1}}*x+b\right)\right] \end{array}$$ (2)

where $k_{\text{1}}$ is the convolution kernel within the channel; b is the bias term of the convolution kernel; $c_{\text{1}}$ is the number of output channels; $F^{\left(1\right)}$ is an output feature composed of $c_{\text{1}}$ channels.

After convolution, the output length within each channel is $L_{\text{1}}$, and the size of feature $F^{\left(1\right)}$ is $\left(c_{\text{1}},L_{\text{1}}\right)$. Then, convolution layers with different kernel sizes perform parallel convolution on the output feature of the previous layer. Specifically, N one-dimensional convolution layers simultaneously convolve feature $F^{\left(1\right)}$. Each convolution layer outputs $c_{\text{2}}$ channels, and the stride of all convolution layers is set to 1. After activation by the ReLU activation function, the outputs are concatenated along the channel axis, the total number of channels is $c_{\text{3}}$ in the final output feature, and $c_{\text{3}}=N\times c_{\text{2}}$. Using convolution kernels of different sizes extracts features at different time scales, greatly enriching the fault feature information. Zero-padding is applied during convolution to ensure that different convolution layers have the same output length $L_{\text{2}}$, so that the output features of different channels can be smoothly concatenated in the concatenation layer. The computational expression for the entire concatenation process is:


$$\begin{array}{c} F_{i}^{\left(2\right)}=f(k_{2i}*F^{\left(1\right)}+b) \end{array}$$ (3)

$$\begin{array}{c} F^{\left(2\right)}={conv}_{N}^{i}\left[F_{i}^{\left(2\right)}\right]\; \end{array}$$ (4)

where $k_{2i}$ is the convolution kernel used by the *i*-th convolutional layer (i=1,2,⋯,N); $F^{\left(1\right)}$ is the input feature; con is the concatenation function; $F_{i}^{\left(2\right)}$ is the feature from the *i*-th convolutional layer, and the size of feature $F^{\left(2\right)}$ is $\left(c_{\text{3}},L_{\text{2}}\right)$.;

### 3.3. Dual attention module

During the extraction of fault discriminative features by CNN, not all features extracted from each convolutional layer contribute positively to fault identification. The extracted features may also contain useless and redundant components, which increase computational cost and interfere with fault diagnosis. The attention mechanism mimics the human information processing workflow of attention allocation, enabling the model to assign distinct weights to different segments of input features for better capture of critical information. Features with higher weights exert a stronger positive impact on model decision-making, while those with lower weights contribute less favorably [26]. The attention module achieves remarkable network performance improvement by introducing only a small number of extra parameters without imposing excessive computational overhead. Therefore, this paper designs the dual attention block. The dual attention module consists of a Channel Attention (CA) branch and a Spatial Attention (SA) branch connected in a cascade manner, following a progressive weighting strategy of “channel screening first, spatial enhancement second”. The CA branch primarily focuses on discriminative information in feature maps, with the Squeeze-and-Excitation Network (SE) as its most representative architecture. It comprises two main components: the squeeze unit and the excitation unit. The squeeze unit compresses and aggregates the global spatial information of feature maps, characterizing the importance of each channel through feature learning in the channel dimension. Subsequently, different weights are assigned to each channel according to the excitation components.

**Fig 3:**
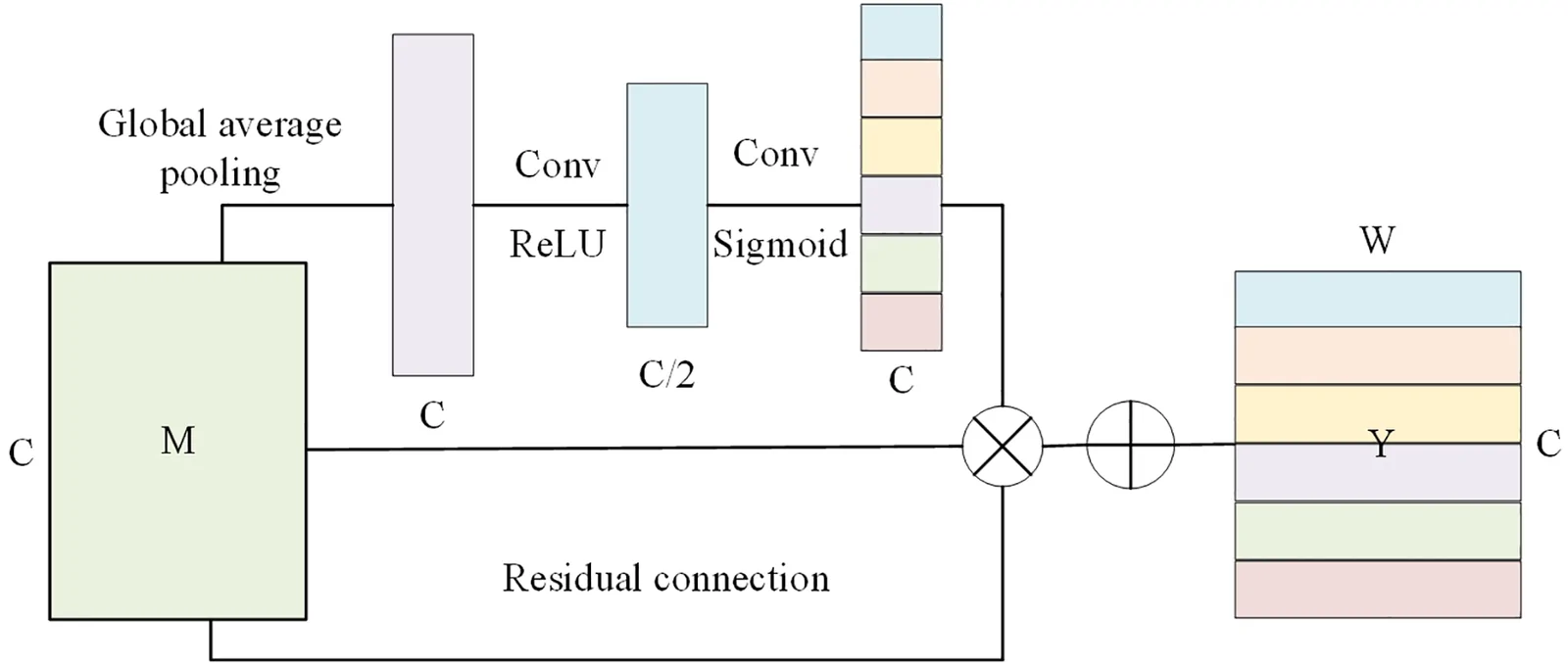
The structure of CA.

The structure of the CA module is illustrated in Fig 3. This module is mainly composed of a global pooling layer, two convolutional layers, a ReLU activation function and a Sigmoid activation function. The global pooling is implemented via average pooling, which serves to compress the dimension of each feature map from H×W×C to 1×1×C in the spatial dimension. Different weights are generated sequentially through convolution operations and activation functions, so as to characterize the importance of each channel [27]. For the input feature set $M=\left\{m_{\text{1}},m_{\text{2}},...m_{i},...,m_{c}\right\}$， $m_{i}\in R^{1\times W}$, the feature map with length *W* is represented. Firstly, the global average pooling of the feature set is changed into $Z\in R^{1\times c}$ to compress the spatial characteristics of each channel, as shown in formula (5).


$$\begin{array}{c} z_{i}=\frac{\text{1}}{1\times W}\sum_{k}^{W}m_{i}\left(k\right) \end{array}$$ (5)

The input feature set M becomes *Z* after space compression, and then becomes $Z^{*}$ through two convolutional layers and activation function. The calculation expression is shown in formula (6).


$$\begin{array}{c} Z^{*}=\delta \left\{F_{\text{2}}\left\{f\left[F_{\text{1}}\left(Z\right)\right]\right\}\right\} \end{array}$$ (6)

where *F*_1_ and *F*_2_ denote convolution operations with a channel number of 1 and a 1×1 convolution kernel size; δ() represents the Sigmoid activation function; $Z^{*}$ represents the importance of each feature channel, and different channels allocate differentiated weight coefficients accordingly. $Z^{*}$ is multiplied by the original input feature set *M* one by one, and the weighted new feature set is obtained, as shown in formula (7).


$$\begin{array}{c} G=\left\{z_{\text{1}}^{*}m_{\text{1}},z_{\text{2}}^{*}m_{\text{2}},...z_{c}^{*}m_{c}\right\} \end{array}$$ (7)

The main idea of residual learning is introduced, and the residual connection structure is embedded in the process of channel attention calculation process, which expands sufficient space for further improvement of model feature expression ability. Its specific calculation formula is as follows:


$$\begin{array}{c} Y=G+M \end{array}$$ (8)

where *Y* is a set of feature sets containing weighted information and original information.

**Fig 4:**
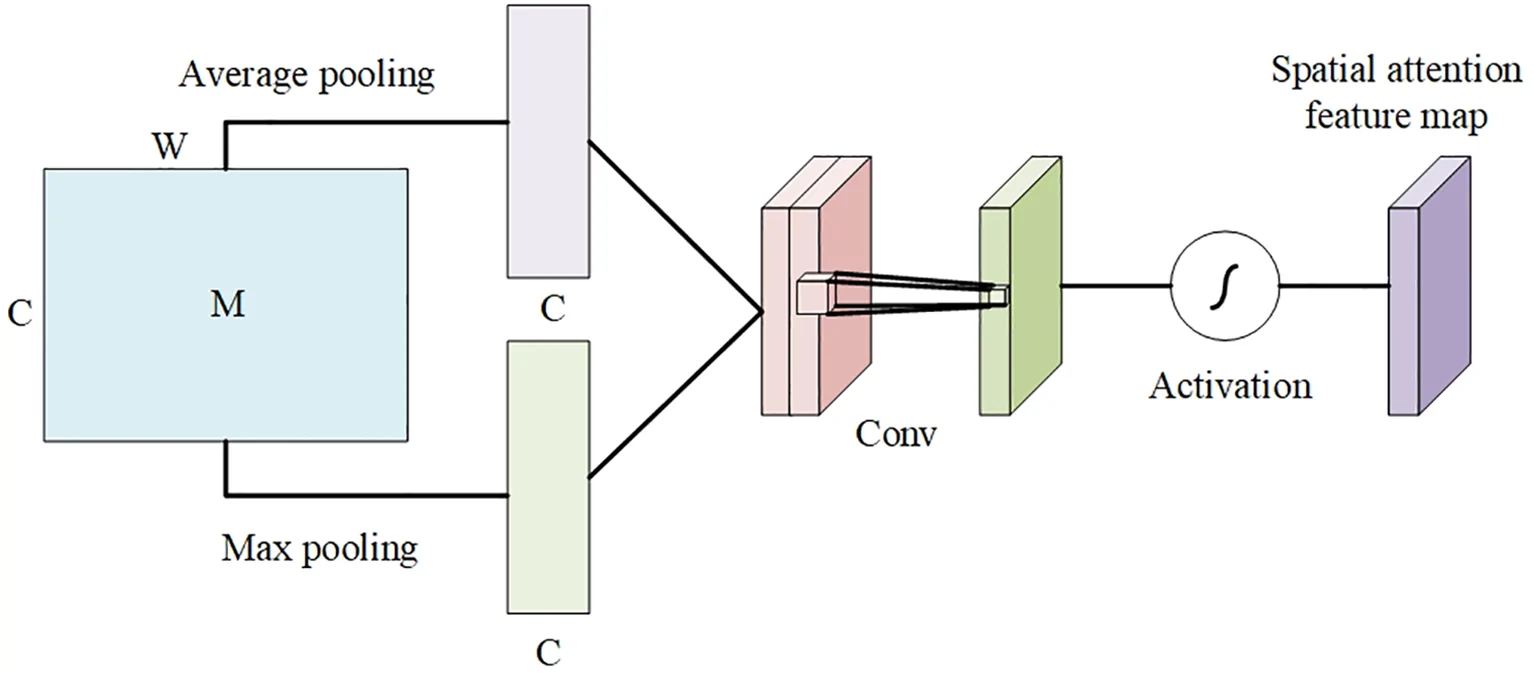
The structure of SA.

Spatial Attention (SA) focuses on the importance of regional information on feature maps along the channel dimension, serving as a complement to CA. The structure of the SA module is shown in Fig 4.

Taking the feature maps output by the CA module as input, this module first performs global max pooling and global average pooling on the input feature maps along the channel dimension to obtain two compressed single-channel feature maps. Afterwards, the outputs of the two pooling operations are concatenated and fused along the channel dimension to generate fused feature maps with a dimension of H×W×2. Finally, through convolution operation and Sigmoid activation function processing, spatial attention weight feature maps with a dimension of H×W×1 are produced [28]. Its calculation formula is given as follows:


$$\begin{array}{c} M_{s}\left(F\right)=f\left\{\tilde{f}\left\{\left[AvgPool\left(F\right);MaxPool\left(F\right)\right]\right\}\right\}=f\left(\tilde{f}\left(\left[F_{avg};F_{max}\right]\right)\right) \end{array}$$ (9)

where $\tilde{f}$ is convolution operation.

**Fig 5:**
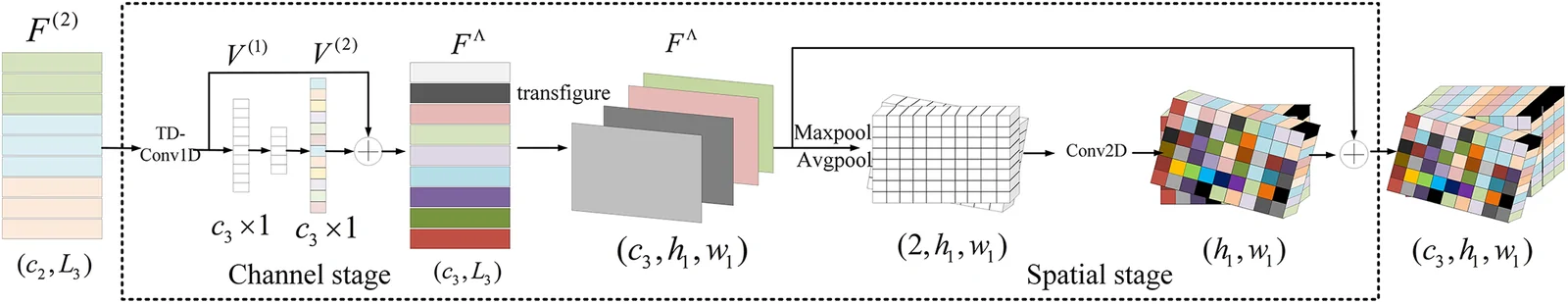
The structure of the two-stage attention module.

Through the multi-scale feature extraction module and the two-stage attention module that performs adaptive weighting modulation on multi-scale features from both the channel and spatial dimensions, the feature extraction capability is enhanced. The structure of the two-stage attention module is illustrated in Fig 5.

In the channel dimension, the module enhances channel features that are more sensitive to bearing health conditions while suppressing those sensitive to working condition variations. In the spatial dimension, it enhances features with stronger fault discriminability within each channel and suppresses useless features. Therefore, this module is a key component for improving the model’s working condition adaptability.

### 3.4. Feature fusion module

**Fig 6:**
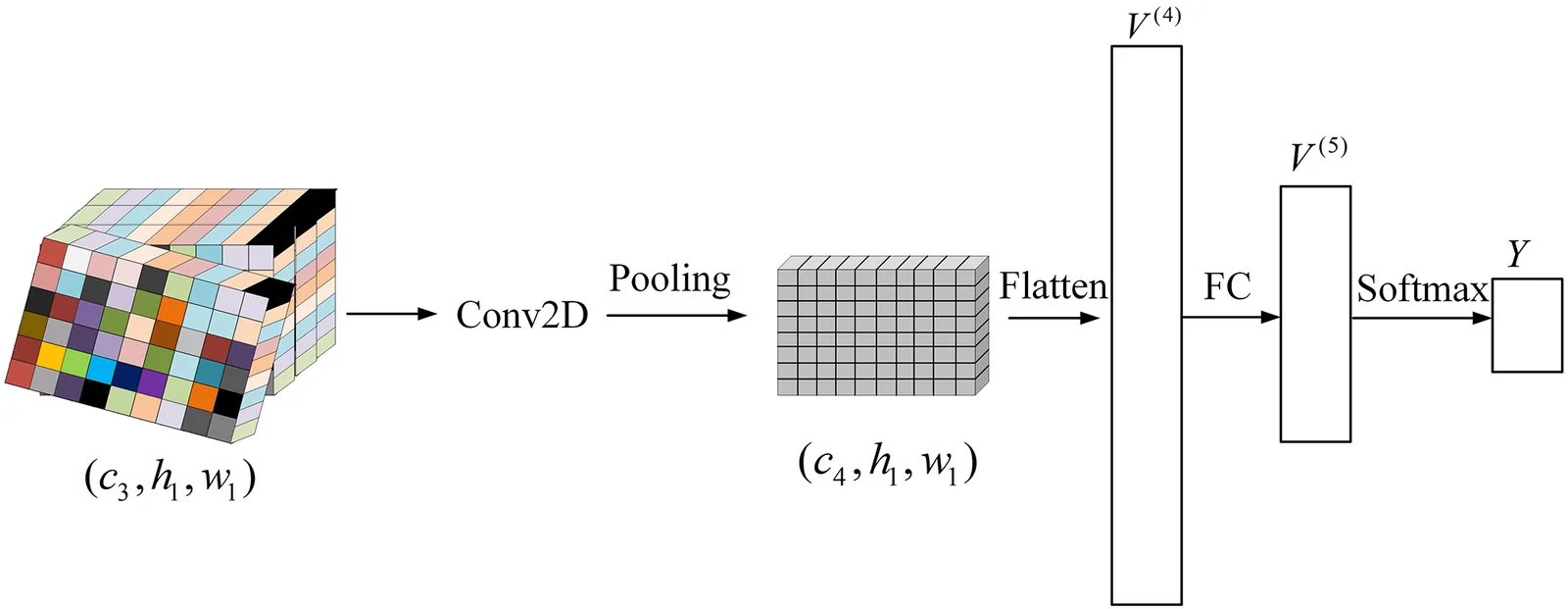
The structure of feature fusion.

As a key connection and integration unit linking the multi-scale feature extraction branches and the two-stage attention weighting module, the feature fusion module performs deep combination, noise elimination, and dimension calibration on the multi-scale features after dual adaptive weighting in the channel and spatial dimensions. It outputs fault feature vectors with strong discriminability and robustness against working condition variations [29]. Through a progressive process including deep feature extraction, large-scale batch normalization, dimension reduction, overfitting suppression, and fully connected layer classification, this module ultimately generates highly robust fault diagnosis results. The structure of the feature fusion module is shown in Fig 6.

As illustrated in Fig 6, the weighted multi-scale features first undergo in-depth global feature extraction, followed by standardization of feature distributions and compression of redundant parameters. Finally, the processed features are converted into one-dimensional feature vectors accessible to the classifier for fault category output. The detailed implementation steps are as follows:

**Step 1:** The weighted feature maps output from the dual attention module are fed into a two-dimensional convolutional layer for secondary global feature mining, enhancing the fused representation of fault information across diverse scales and channels. Channel-wise convolution is performed using small-size convolution kernels, which preliminarily aggregates and regularizes multi-scale feature dimensions while maintaining complete fault feature information. A batch normalization layer is deployed after each two-dimensional convolution operation to alleviate internal covariate shift during the training of deep neural networks, accelerate model convergence, avoid diagnostic accuracy fluctuations caused by inconsistent feature distributions, and improve model stability under variable working conditions.

**Step 2:** Max pooling layers are adopted to downsample the normalized features, retaining salient fault features in feature maps and removing redundant information. A Dropout regularization layer is introduced after pooling to greatly reduce the risk of model overfitting.

**Step 3:** The two-dimensional feature maps output by Dropout are flattened into one-dimensional feature vectors through the Flatten layer. This converts high-dimensional features into vector formats processable by fully connected layers and completes the format transformation from spatially distributed features to classification vectors.

**Step 4:** Feature prediction is implemented via fully connected layers and the Softmax classifier. The fully connected layers conduct nonlinear mapping and transformation on one-dimensional feature vectors to further integrate global features and output feature vectors whose dimensions match the total number of bearing fault categories. The Softmax classifier normalizes the outputs of the fully connected layers into a probability distribution within the range [0,1], where each probability value corresponds to one health state of the bearing. The health state corresponding to the maximum probability in the distribution is selected as the final fault diagnosis result output by the model.

## 4. Experimental results and analysis

In this paper, the publicly available rolling bearing fault dataset released by Case Western Reserve University (CWRU) is selected as the benchmark dataset for experimental verification. As one of the international standard benchmark datasets, it enjoys the highest recognition and is most widely applied in the field of rolling bearing fault diagnosis. The CWRU bearing fault experimental platform mainly consists of a drive motor, a torque sensor, a power tester, an electronic control system, and accelerometers installed at the drive end and fan end of the motor, enabling the collection of original bearing vibration signals under different working conditions. Vibration acceleration signals are collected from SKF 6205 deep groove ball bearings mounted at the motor drive end under three load conditions: 0.75 kW, 1.50 kW, and 2.25 kW. Single-point damage defects are prefabricated on key load-bearing components, namely the rolling elements, inner race, and outer race, using electrical discharge machining (EDM). The dataset covers four typical health states of rolling bearings: normal operation, inner race fault, outer race fault, and rolling element fault. For each fault type, three damage severity levels are set with damage diameters of 0.007 mm, 0.014 mm, and 0.021 mm, respectively. Accordingly, each load condition involves a total of 10 distinct bearing health states, derived from 1 normal state plus 3 fault types × 3 damage severity levels. In the experiment, the vibration signal of each sample is intercepted into data segments with a length of 2000 sampling points. The dataset under each load is divided into a training set (1200 samples), a test set (300 samples) and a validation set (1500 samples) at a ratio of 4:1:5. The detailed division of the experimental dataset is shown in Table 1.

**Table 1:** Bearing fault classification and label value.

Label value	Fault type	Fault diameter/inch
0	Normal state	nil
1	Inner ring fault	0.007
2	Rolling element fault	0.007
3	Outer race defect	0.007
4	Inner ring fault	0.014
5	Rolling element fault	0.014
6	Outer race defect	0.014
7	Inner ring fault	0.021
8	Rolling element fault	0.021
9	Outer race defect	0.021

### 4.1. Evaluation metrics

To comprehensively and objectively evaluate the performance of the network model in rolling bearing fault diagnosis, accuracy is adopted as the core evaluation metric to assess the overall diagnostic performance. Meanwhile, recall and F1-score are employed as supplementary evaluation metrics to comprehensively measure the model’s fault recall capability and classification precision. The calculation formulas of the above evaluation metrics are expressed as follows:


$$\begin{array}{c} \left\{ \begin{array}{c} \text{Accuracy}=\frac{\text{TP}+\text{TN}}{\text{TP}+\text{TN}+\text{FP}+\text{FN}}\\ \text{Recall}=\frac{\text{TP}}{\text{TP}+\text{FN}}\\ F1-score=2*\frac{Accuracy\times Recall}{Accuracy+Recall} \end{array}\right. \end{array}$$ (10)

where TP, TN, FP and FN denote the number of true positives, true negatives, false positives and false negatives, respectively. The detailed information of different working conditions is shown in Table 2.

**Table 2:** Information of different working conditions.

No.	Working information
Working condition 1	0.75KW/1772r/min
Working condition 2	1.50KW/1750r/min
Working condition 3	2.25KW/1730r/min

### 4.2. Fault diagnosis results and analysis under variable working conditions

**Fig 7:**
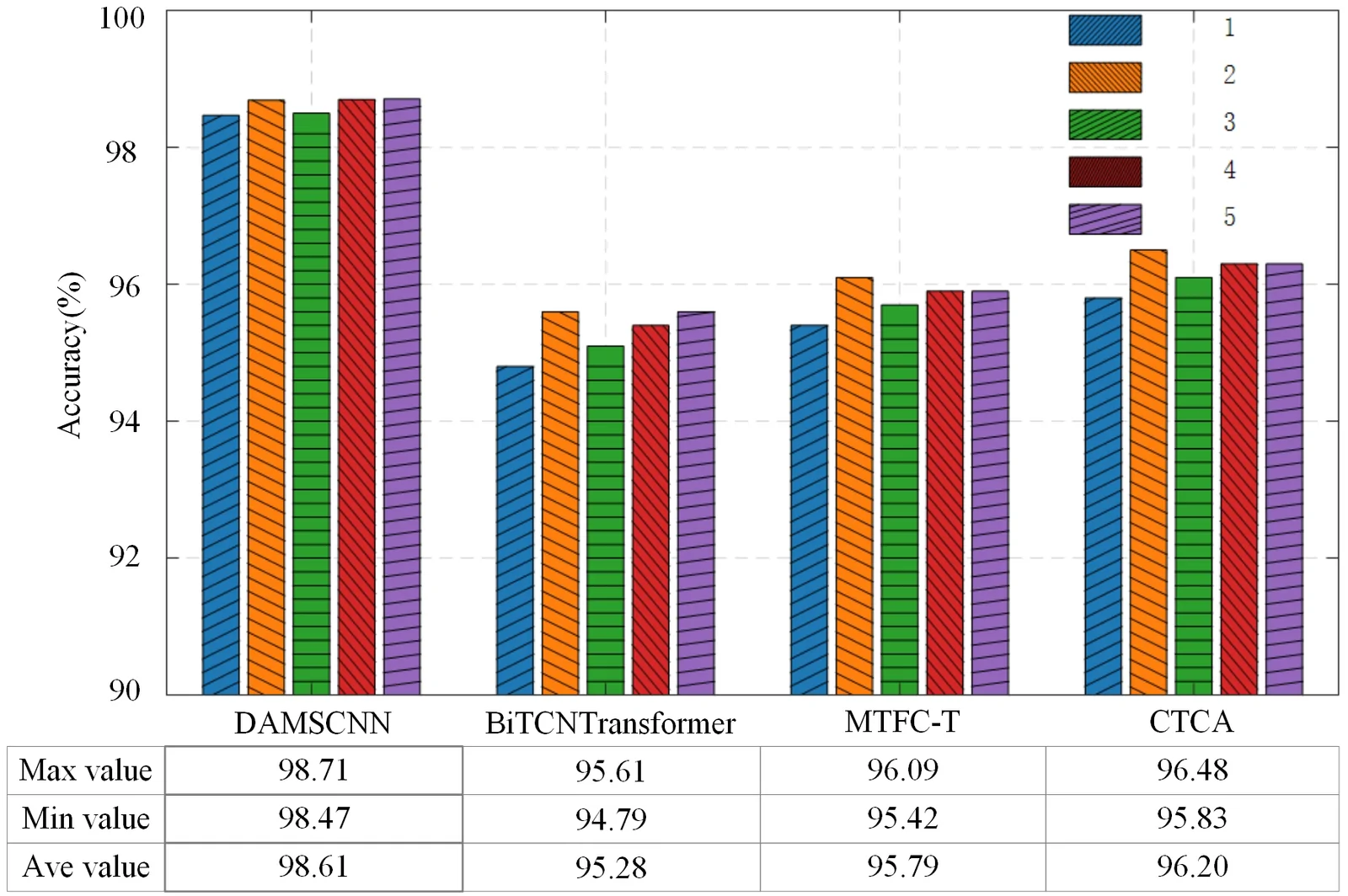
Diagnostic accuracy of models when the operating condition changes from working condition 1 to working condition 2.

In this paper, bearing equipment is selected as the research object, and different load conditions are adopted to simulate scenarios of varying working conditions. Bearing signals under motor load A are taken as the source working condition data, while those under load B serve as the target working condition data. The specific parameter configuration of each task is listed in Table 3. To comparatively evaluate the performance of different network models under variable working conditions, comparative fault diagnosis experiments are conducted based on the BiTCN-Transformer, MTFC-T, CTCA, and DAMSCNN models, with the corresponding results presented in Table 4. In order to ensure the accuracy of the experimental results, each model repeats the experiment 5 times, for example, when the operating condition changes from Condition 1 to Condition 2, Fig 7 presents the test results. To comprehensively evaluate the performance and computational overhead of DAMSCNN in bearing fault diagnosis tasks, we conducted a detailed analysis using parameters (Params), computational complexity (FLOPs), and training time (T), and compared it with BiTCN-Transformer, MTFC-T, and CTCA. The results are presented in Table 5.

**Table 3:** Variable working condition task description.

Variable working conditions	Source working condition	Target working condition
1-2	0.75KW/1772r/min	1.50KW/1750r/min
1-3	0.75KW/1772r/min	2.25KW/1730r/min
2−1	1.50KW/1750r/min	0.75KW/1772r/min
2-3	1.50KW/1750r/min	2.25KW/1730r/min
3−1	2.25KW/1730r/min	0.75KW/1772r/min
3−2	2.25KW/1730r/min	1.50KW/1750r/min

**Table 4:** Experimental results in the variable working conditions.

Variable working conditions	BiTCNTransformer	MTFC-T	CTCA	DAMSCNN
1-2	95.28	95.79	96.20	98.61
1-3	96.02	95.23	95.69	99.18
2−1	94.48	95.77	95.36	98.42
2-3	94.92	96.20	94.93	99.69
3−1	93.29	97.21	95.92	98.64
3−2	95.36	96.58	96.23	97.91

**Table 5:** Computational complexity analysis of different models.

Model	Params(M)	FLOPS(M)	T(ms/sample)
BiTCNTransformer	0.75	50	10 ~ 15
MTFC-T	0.46	35	8 ~ 10
CTCA	0.54	55	12 ~ 20
DAMSCNN	12.7	25	4 ~ 6

**Fig 8:**
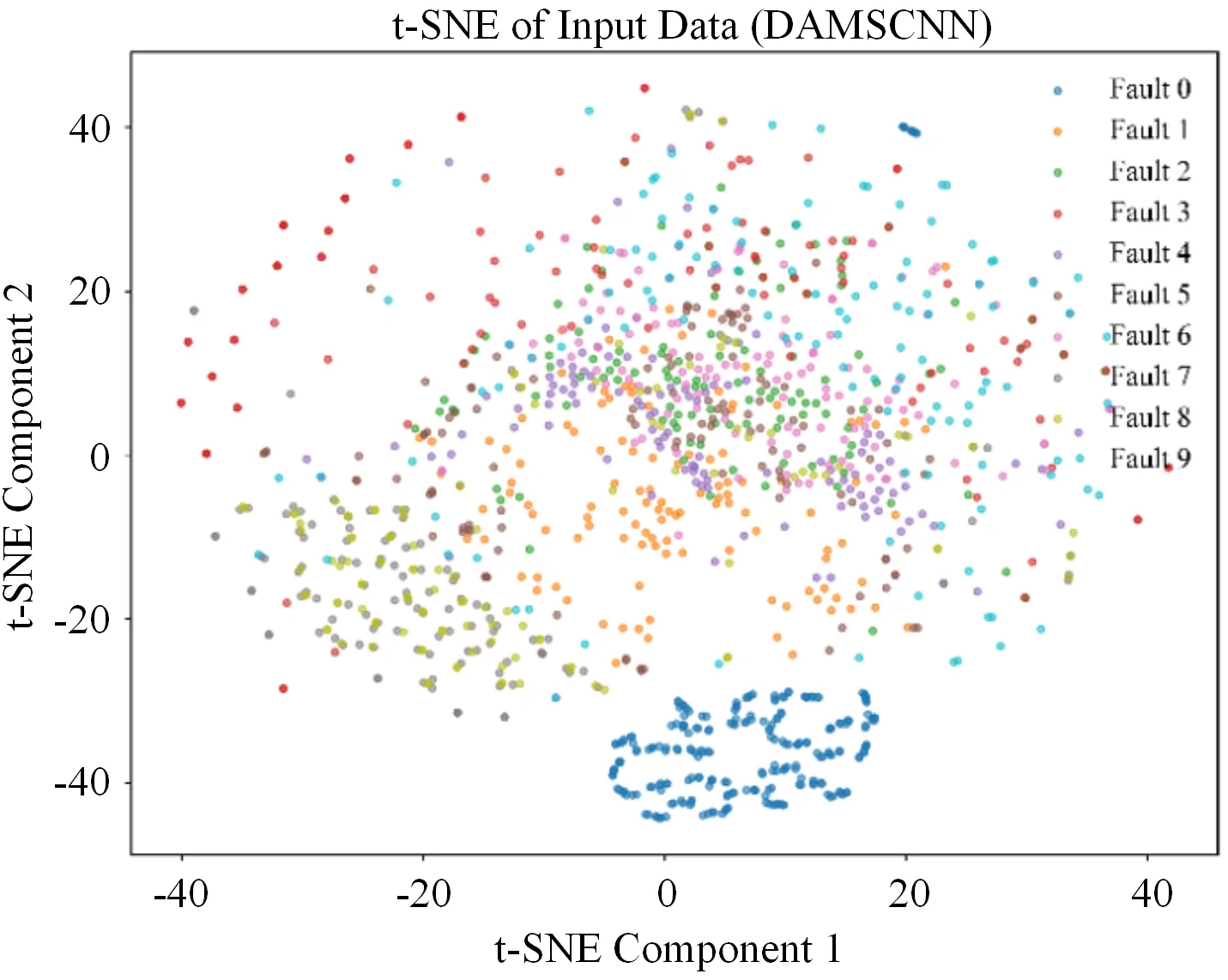
The t-SNE feature distribution under variable working conditions(Feature distribution at the input end).

As can be seen from the results in Table 4, the proposed DAMSCNN model achieves significantly higher diagnostic accuracy than the three comparative models across all cross-condition transfer tasks, with accuracy exceeding 97.91% in every task. Specifically, the model reaches its highest diagnostic accuracy of 99.69% in the transfer task from working condition 2 to working condition 3. In contrast, the diagnostic accuracies of BiTCNTransformer, MTFC-T and CTCA range from 93% to 97%. For example, BiTCNTransformer only achieves an accuracy of 93.29% in the transfer task from working condition 3 to working condition 1, indicating its weak cross-condition adaptation capability. Although MTFC-T and CTCA obtain certain generalization performance owing to the global modeling ability of Transformer, their diagnostic accuracy still fluctuates notably under working condition variations. Taking the transfer task from working condition 1 to working condition 2 as an example, Fig 8 shows that DAMSCNN has a significantly higher average accuracy than the other comparative models, with lower dispersion of results across repeated experiments. This demonstrates stable training convergence of the model and favorable reproducibility of the diagnostic results.

As presented in Table 5, in terms of parameter count, DAMSCNN has a total of 12.7M parameters, which is larger than that of the other three comparative models. However, from the perspective of computational efficiency, DAMSCNN only requires 25M floating-point operations (FLOPs), which is remarkably lower than those of BiTCNTransformer (50M), MTFC-T (35M) and CTCA (55M). In addition, the single-task inference time of DAMSCNN is only 4–6 s, and its inference speed is more than twice that of the other comparative models.

**Fig 9:**
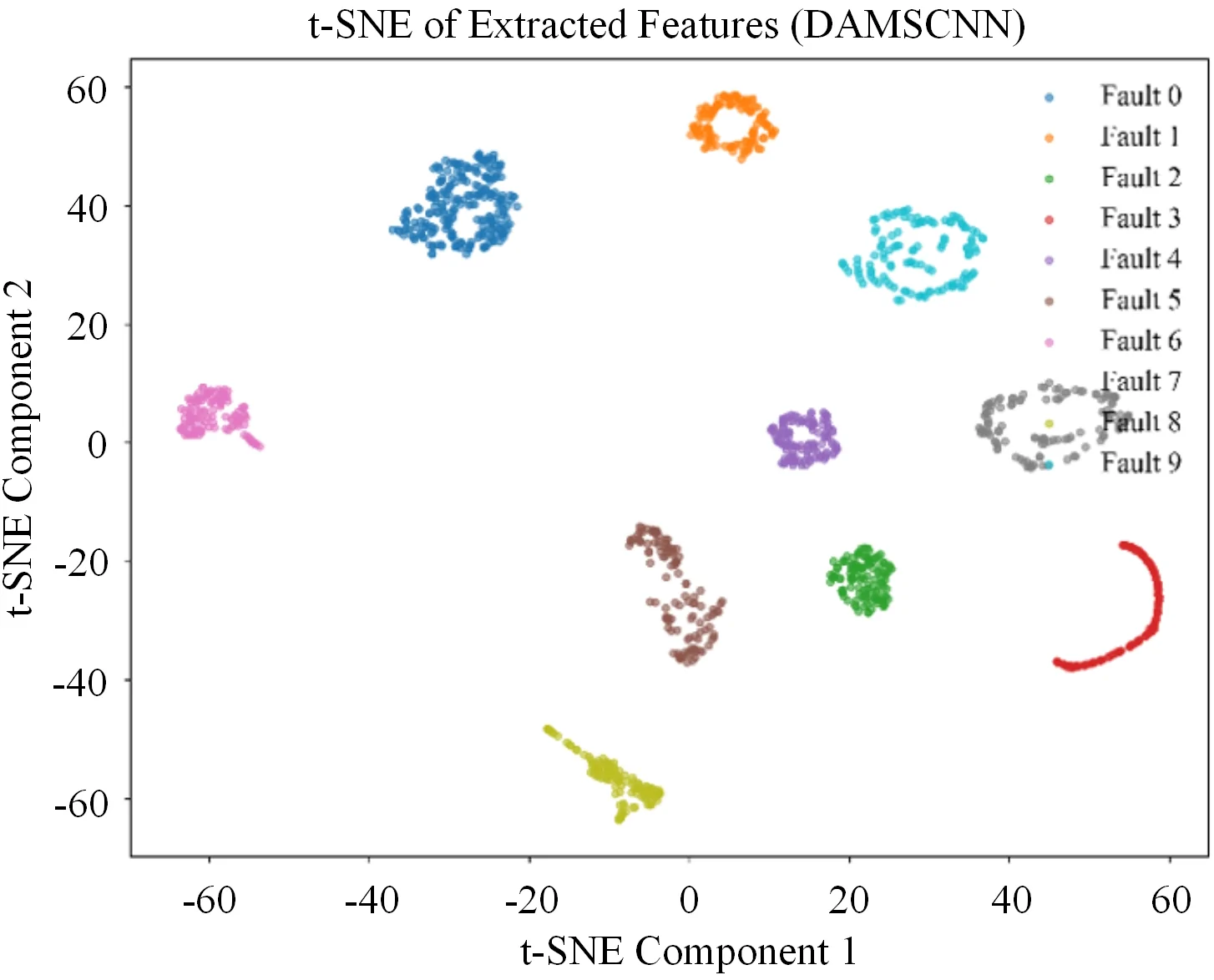
The t-SNE feature distribution under variable working conditions(Feature distribution at the output end).

The t-SNE dimensionality reduction visualization method is adopted to verify the feature extraction performance of the DAMSCNN model under variable working conditions. Fig 8 presents the t-SNE feature distribution results for the transfer scenario from working condition 1 to working condition 2, where Fig 8 shows the feature distribution at the input end and Fig 9 shows that at the output end.

As can be seen from Fig 9, the target working condition fault features extracted by the DAMSCNN model achieve highly compact clustering of intra-class samples and effective differentiation of inter-class samples. Samples of each category are densely distributed with almost no discrete outliers. This demonstrates that under variable working conditions, DAMSCNN can effectively strip out working condition interference information and accurately mine and extract highly discriminative fault features, which fully reflects the model’s excellent cross-condition transfer capability and sound generalization performance.

### 4.3. Fault results and analysis under strong noise conditions

During on-site industrial operation, rolling bearings frequently work under complex and variable working conditions. Consequently, the collected vibration signals are easily contaminated by various types of electromagnetic noise, mechanical impact noise, and background noise in the industrial environment, and are also interfered with by them. This results in weak vibration signals and submerges effective fault features in background noise, thereby increasing the difficulty and complexity of fault diagnosis. To quantitatively evaluate the relative intensity of signals and noise, the signal-to-noise ratio (SNR) is adopted as the indicator, and its calculation expression is as follows:


$$\begin{array}{c} S_{NR}=lg\frac{P_{signal}}{P_{noise}} \end{array}$$ (11)

**Fig 10:**
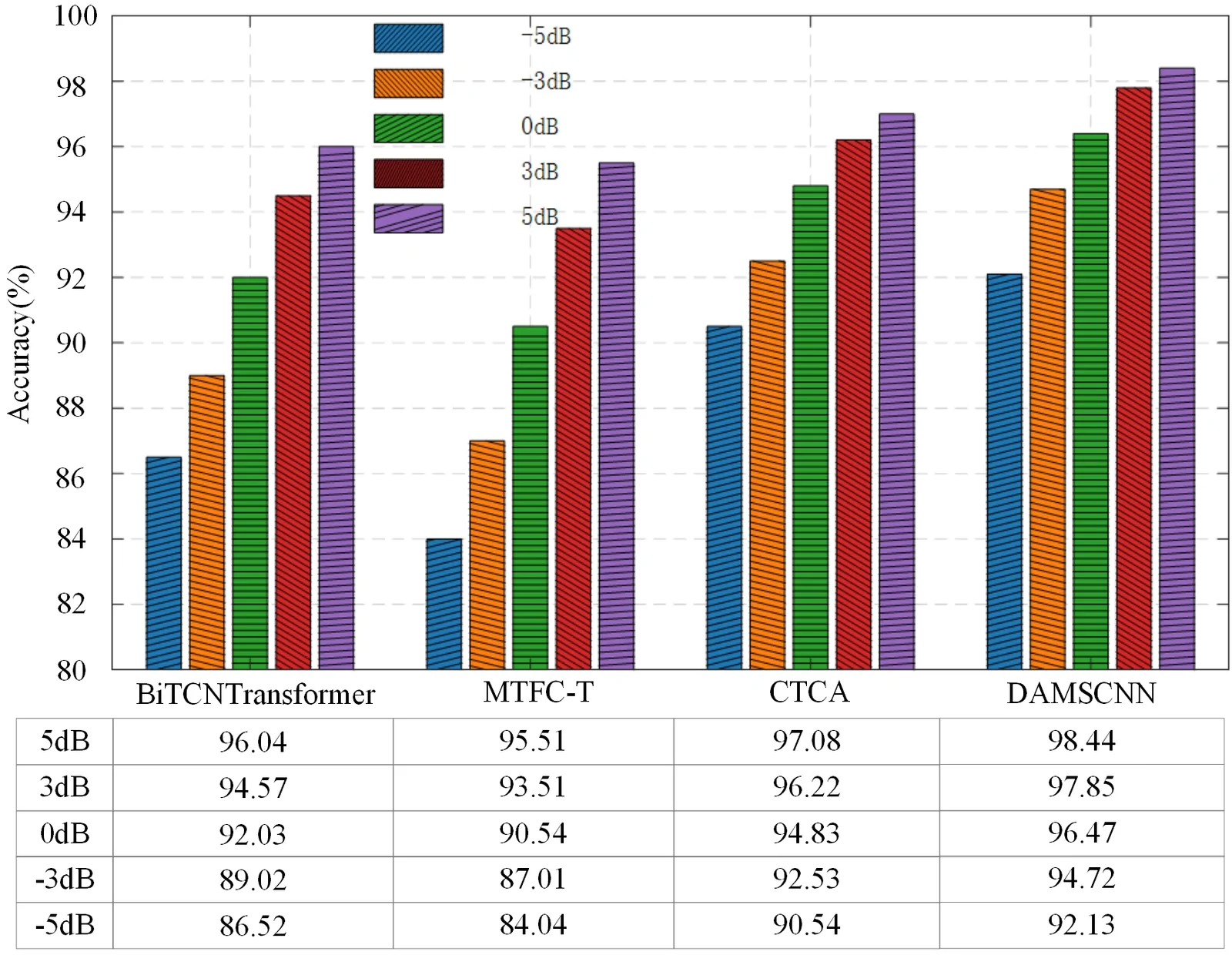
Diagnostic performance under different signal-to-noise ratios(Accuracy).

**Fig 11:**
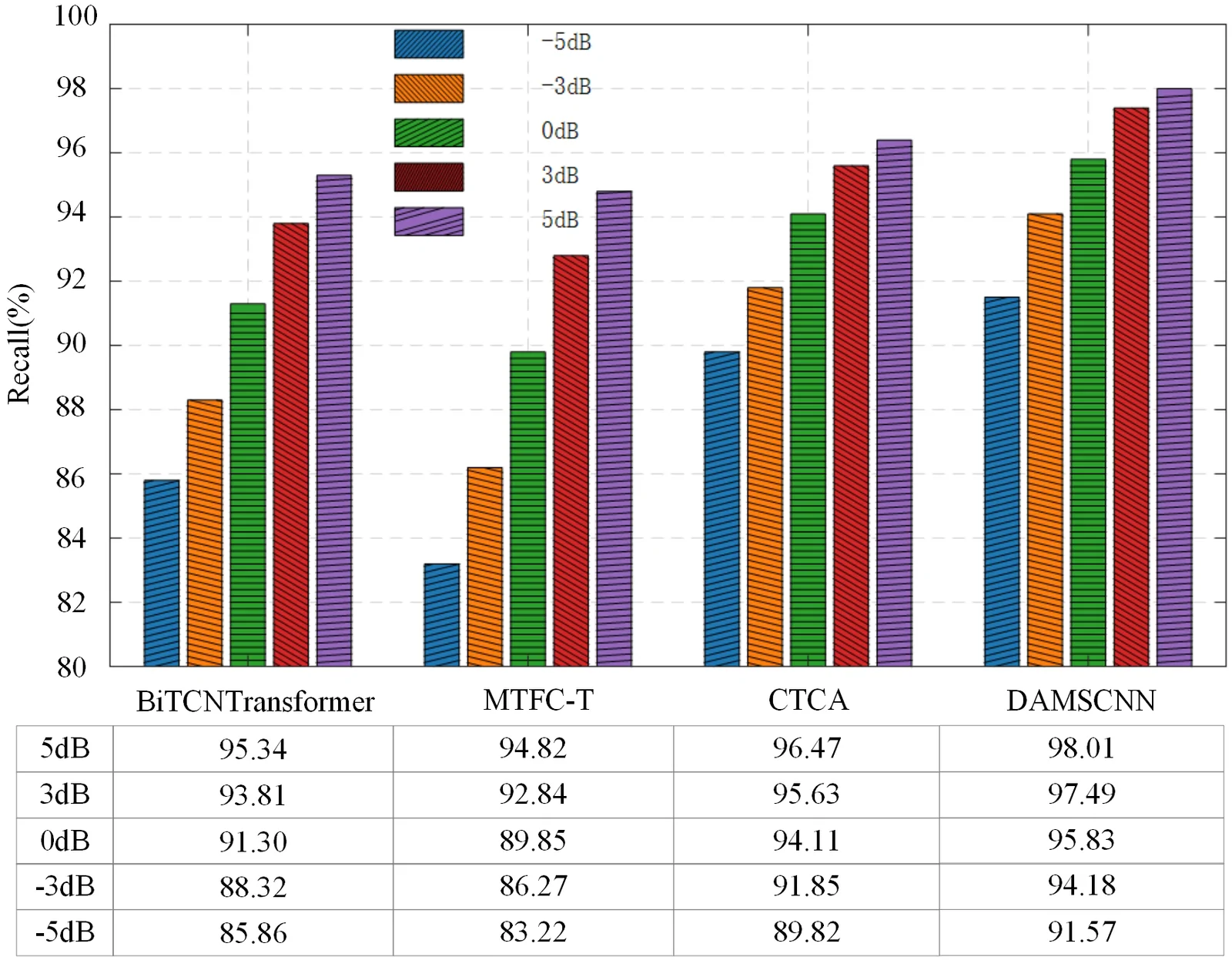
Diagnostic performance under different signal-to-noise ratios(Recall).

**Fig 12:**
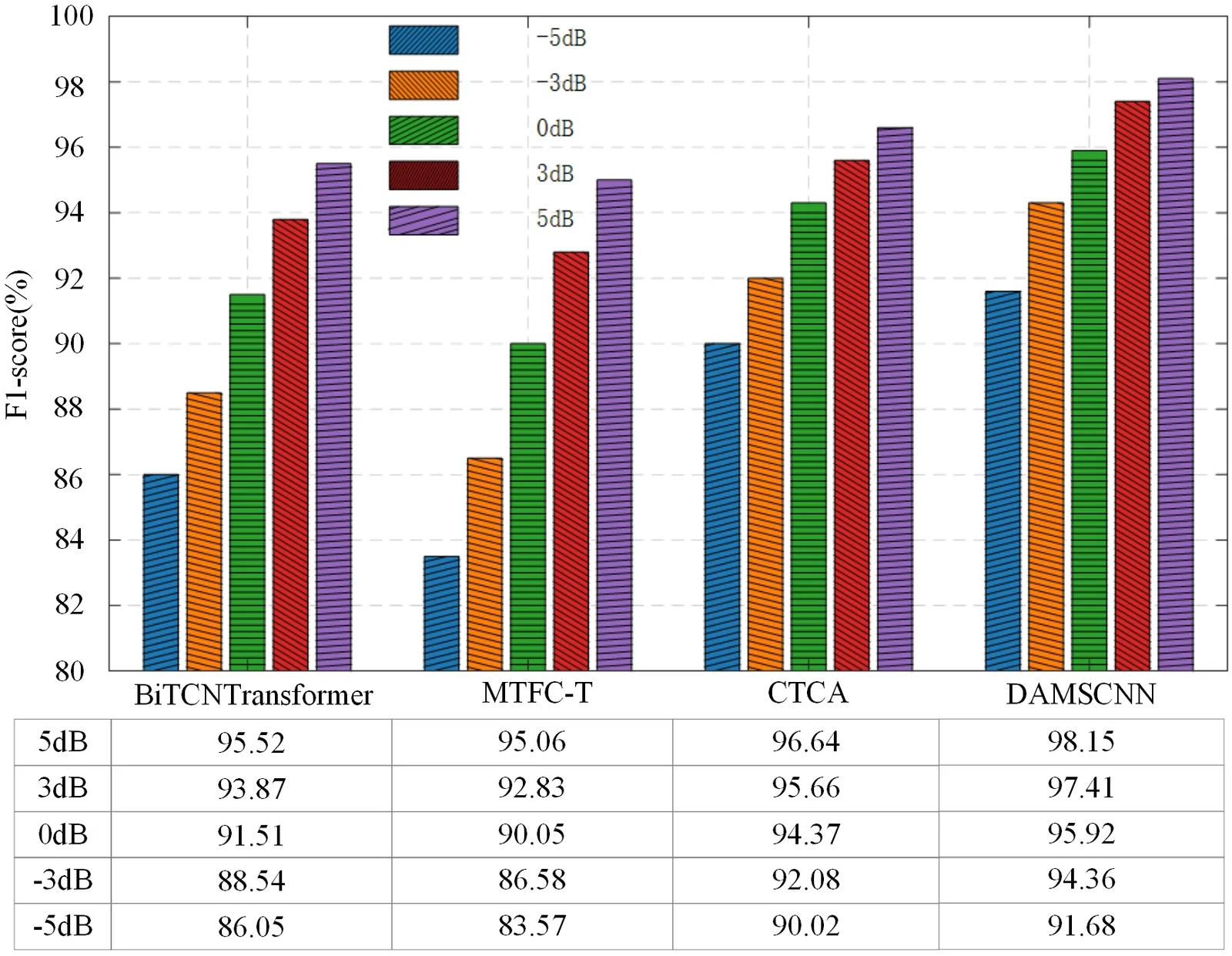
Diagnostic performance under different signal-to-noise ratios(F1-score).

Gaussian white noise with SNRs of −5, −3, 0, 3 and 5 dB is added to the dataset to conduct fault diagnosis experiments under simulated strong noise conditions. The performance metrics of the proposed DAMSCNN model under different SNRs are presented in Fig 9. As can be observed from Fig 10, Fig 11, Fig 12, with the decrease of SNR and the increase of noise interference intensity, the fault impulse features in vibration signals are gradually submerged by background noise. The accuracy, recall and F1-score of all models decline to varying degrees, which reflects the universal negative impact of noise interference on fault diagnosis performance. Under the same SNR condition, the accuracy, recall and F1-score of DAMSCNN are all significantly higher than those of the three comparative models, namely BiTCNTransformer, MTFC-T and CTCA. Particularly in strong noise scenarios with low SNR, DAMSCNN exhibits more prominent performance advantages: it not only maintains high fault diagnosis accuracy, but also keeps recall and F1-score at favorable levels. This indicates that the model effectively suppresses missed detection and misclassification of various faults, and possesses stable and reliable comprehensive fault identification capability.

**Fig 13:**
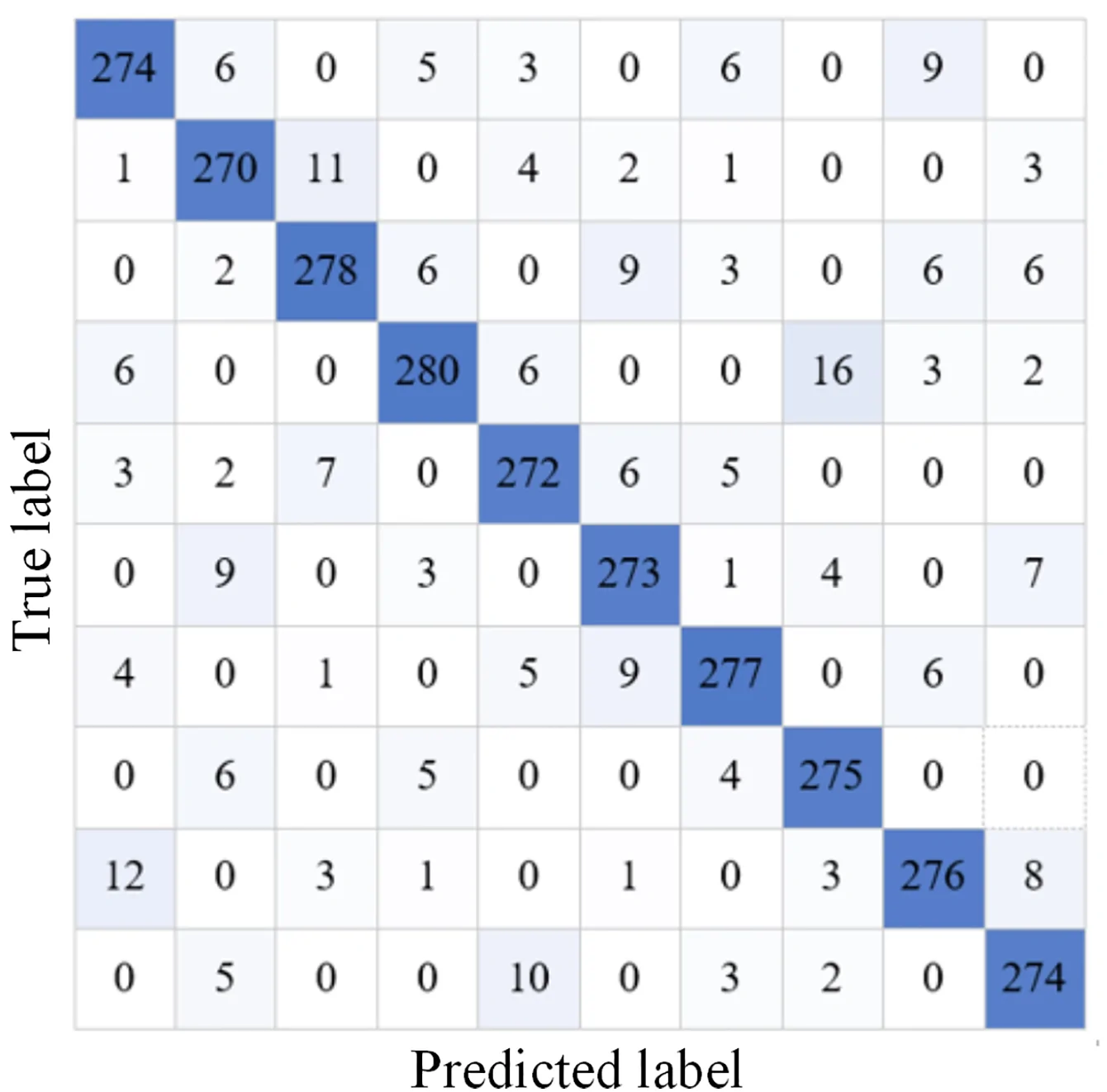
The fault diagnosis confusion matrix of the DAMSCNN model under a noise environment with an SNR of −5 dB.

Fig 13 presents the fault diagnosis confusion matrix of the DAMSCNN model under a noise environment with an SNR of −5 dB.

As can be observed from Fig 13, under the −5 dB noise condition, the diagnostic accuracy of the DAMSCNN model for all nine types of bearing faults is no less than 92%. Each fault category is clearly distinguished with negligible inter-class confusion, reflecting the model’s stable and reliable fault diagnosis performance in strong noise environments.

**Fig 14:**
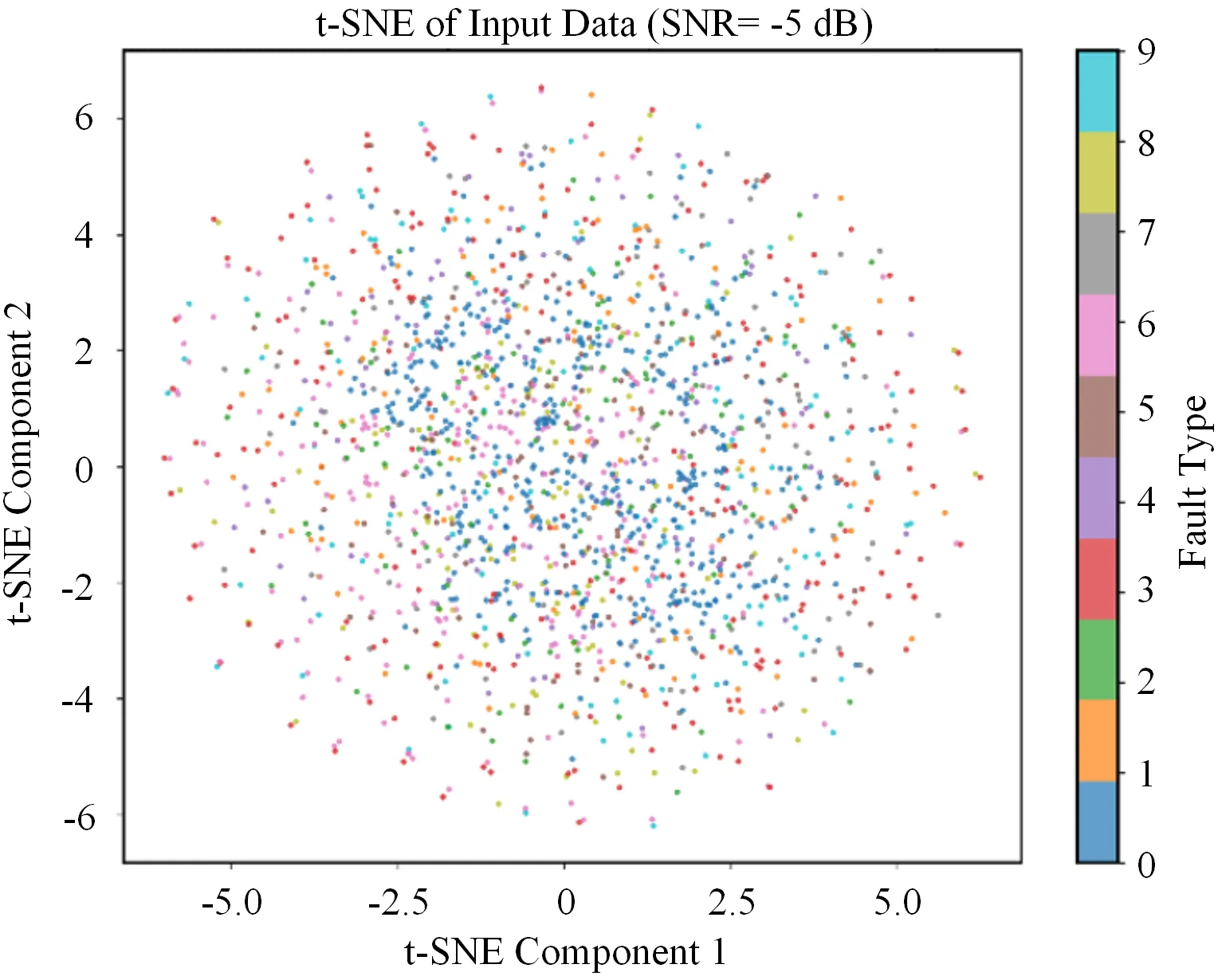
The t-SNE visualization results under the −5 dB SNR noise condition(Feature distribution at the input end).

**Fig 15:**
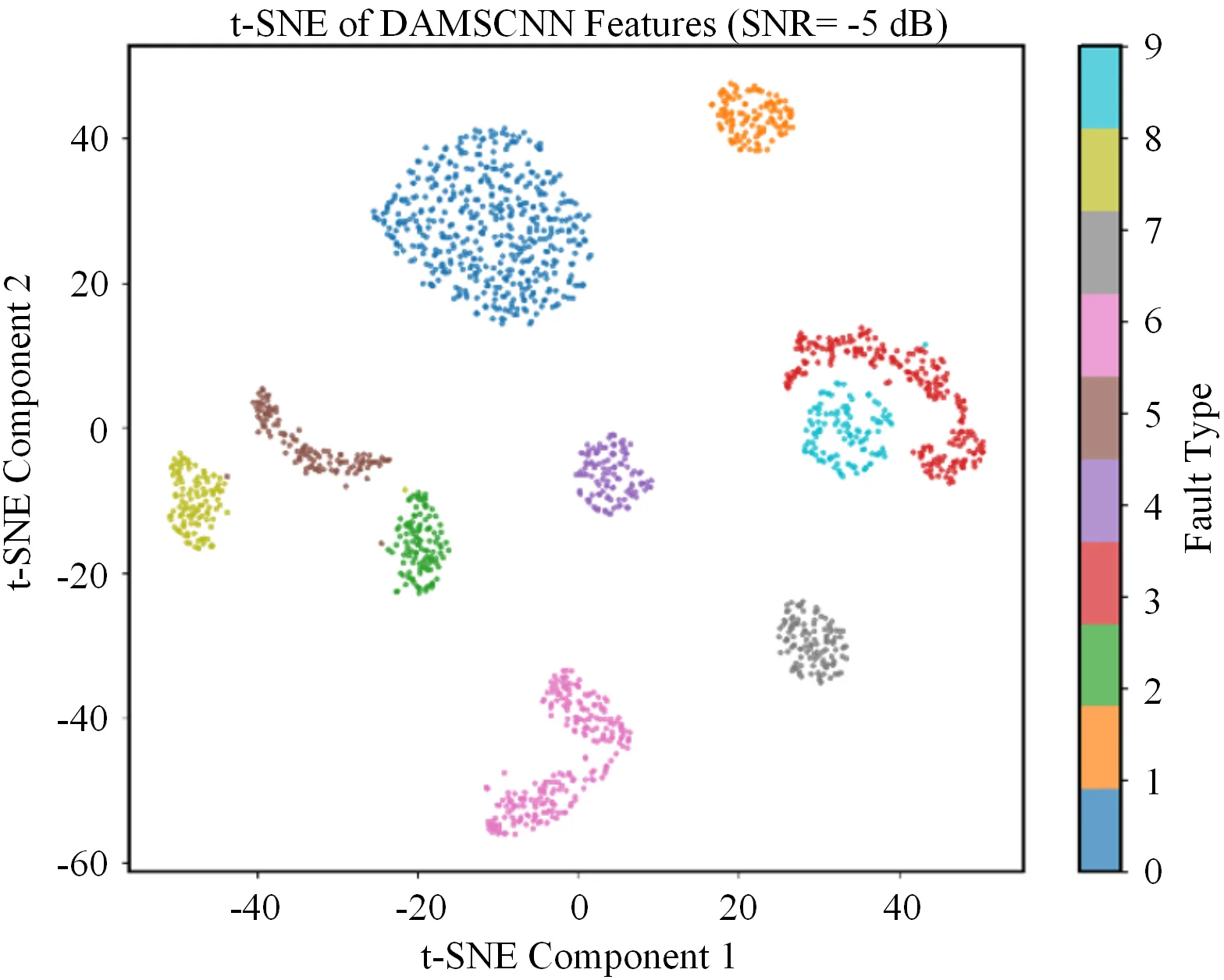
The t-SNE visualization results under the −5 dB SNR noise condition(Feature distribution at the output end).

Fig 14−15 illustrates the t-SNE visualization results of the features extracted by the DAMSCNN model under the −5 dB SNR noise condition, where Fig 14 shows the feature distribution at the input end and Fig 15 shows that at the output end.

As can be seen from Fig 15, after being processed by the DAMSCNN model, fault samples of different types present prominent intra-class clustering and inter-class separation, with no noticeable mixing or dispersion.

### 4.4. Ablation experiment

To verify the role of each module in the model, CNN is adopted as the baseline network, and the results of the ablation study are presented in Table 6. Model 1 incorporates only the multi-scale convolution module. Model 2 incorporates both the multi-scale convolution module and the dual attention module. Model 3 incorporates the dual attention module and the feature fusion module.

**Table 6:** Ablation experiment.

Module	Multi-scale convolution	Spatial attention	Channel attention	Feature fusion	Accuracy(%)
Model 1	√				95.97
Model 2	√	√	√		96.21
Model 3		√	√	√	96.52
DAMSCNN	√	√	√	√	99.89

As can be seen from the experiment results in Table 6, Model 1, which only incorporates the multi-scale convolution module, achieves the accuracy of 95.97%, delivering a certain degree of performance improvement over the baseline CNN model. This verifies that the multi-scale feature extraction structure can effectively capture multi-scale fault impulse information in bearing vibration signals. On this basis, after further cascading the dual attention module, the accuracy of Model 2 rises to 96.21%. This result indicates that the dual attention mechanism screens high-value feature channels and suppresses redundant interference components through adaptive weight allocation along the channel dimension, and highlights key regions of fault impulses via weight mapping in the spatial dimension, effectively enhancing the fault discriminative capability of features. Model 3, equipped with only the dual attention module and the feature fusion module, attains the accuracy of 96.52%. This demonstrates that the feature fusion module performs deep integration of the weighted multi-dimensional features through batch normalization, pooling dimensionality reduction and fully connected mapping, so as to further mine the intrinsic fault correlation information embedded in features and improve classification discrimination performance. When the three modules are fully integrated into the proposed DAMSCNN model, the accuracy reaches 99.89%. This remarkable performance leap indicates that the core modules are not a simple superposition of functions; instead, they constitute a complete processing pipeline of “multi-scale feature extraction - dual-dimensional adaptive weighting and enhancement - deep feature fusion and classification”. The three modules complement each other functionally and generate synergistic effects, ultimately yielding a substantial improvement in fault diagnosis performance, which fully validates the rationality and scientific validity of the proposed model architecture.

## 5. Conclusion

To address the issue of low diagnostic accuracy of rolling bearings caused by variable working conditions and strong background noise in industrial sites, this paper proposes a novel rolling bearing fault diagnosis method named DAMSCNN, which integrates a two-stage cascaded channel-spatial attention mechanism into a multi-scale convolutional architecture. The parallel multi-scale convolution module achieves comprehensive capture of multi-scale fault features through multi-size receptive fields, while the channel-spatial dual attention mechanism adaptively enhances discriminative fault features and suppresses interference from working condition fluctuations and noise. Working in coordination with the feature fusion module, these core components collectively improve the model’s cross-condition generalization performance and noise robustness. The main conclusions drawn from experimental verification are as follows:

(1) In cross-load variable-condition diagnostic tasks, DAMSCNN achieves optimal diagnostic performance among all compared models, with a peak accuracy of 99.69%, verifying the excellent cross-domain generalization capability of the proposed method.
(2) In the noise robustness test across the SNR range of −5 dB to 5 dB, DAMSCNN maintains stable and superior diagnostic performance. Under the most severe noise condition (−5 dB), accuracy still exceeds 92%, with both recall and F1-score remaining at a high level, indicating that the model possesses strong anti-interference capability.
(3) Ablation studies confirm the individual contributions of each core module and synergistic effects between modules. The DAMSCNN model achieves accuracy of 99.89%, validating the rationality and scientific validity of the proposed multi-module fusion architecture. Meanwhile, computational complexity analysis demonstrates that DAMSCNN exhibits application potential for real-time industrial condition monitoring deployment.

Nevertheless, this study still has some limitations. In future research, we will cooperate with industry partners to collect field-measured bearing vibration data with accurate fault labels under real working conditions and conduct experimental verification to further improve the engineering applicability of the proposed method. At the same time, we will construct more realistic industrial noise simulation scenarios and explore fusion methods of noise suppression and fault feature enhancement to strengthen the model’s generalization performance in real complex noise environments.
